# Amelanotic Cellular Blue Nevus: An Unusual Iris Localization

**DOI:** 10.1155/2012/209603

**Published:** 2012-08-16

**Authors:** Silvana Guerriero, Lorenza Ciracì, Tiziana Tritto, Maria Grazia Fiore, Domenico Piscitelli

**Affiliations:** ^1^Department of Neurosciences and Sense Organs, University of Bari, Piazza Giulio Cesare 11, 70124 Bari, Italy; ^2^Department of Pathological Anatomy, University of Bari, Piazza Giulio Cesare 11, 70124 Bari, Italy

## Abstract

The authors describe the first case of eye amelanotic cellular blue nevus reported in literature and discuss the main differential diagnosis.

## 1. Introduction

The blue nevus is a benign, usually solitary lesion which is characterized by a localized proliferation of melanocytes and is believed to represent an abnormal arrest in embryonal migration of neural crest melanocytes [1].

Blue nevi are most commonly found on the skin. Rare cases of common blue nevi have been reported in the vagina [2], the uterine cervix [3], the Mullerian tract [4], the spermatic cord [5], the oral mucosa [6, 7], the prostate [8], and the bronchus [9] in the brain [10] and in the lymph node [11]. No eye localizations are described in literature.

We report an unusual case of iris amelanotic cellular blue nevus in a young woman.

## 2. Case Report

**Figure 1:**
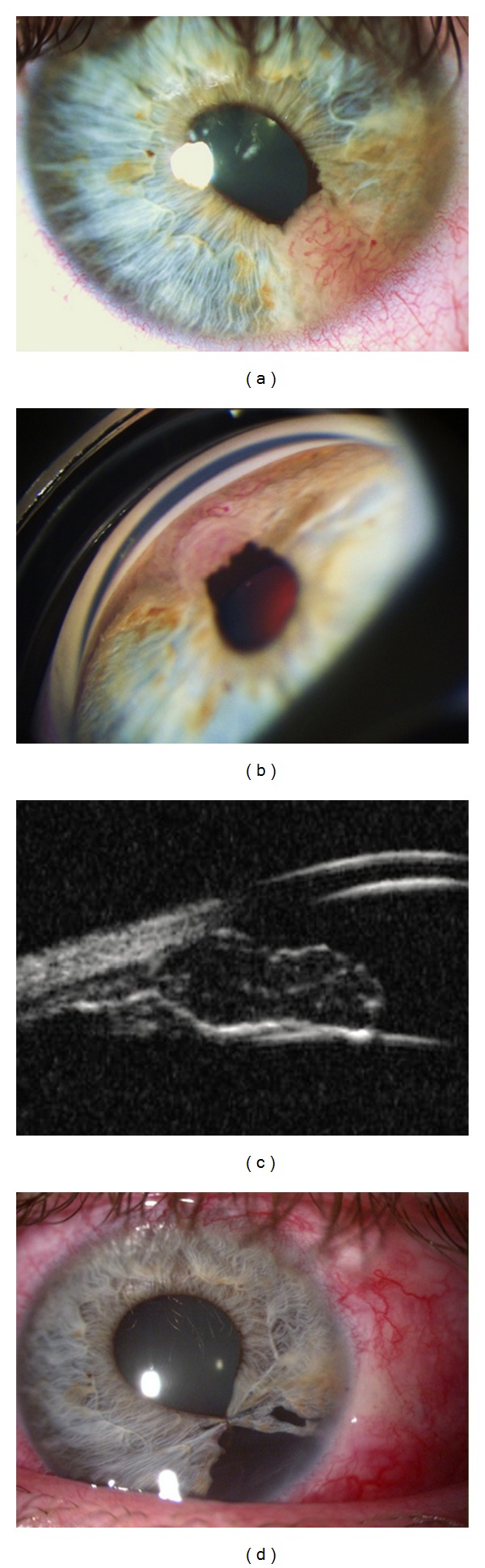
(a) Anterior segment of left eye showing an iris neoformation in the inferior temporal quadrant, richly vascularized. (b) Gonioscopic appearance of the neoformation. (c) UBM image showing a low reflectivity and poorly demarcated neoformation invading the iris stroma. (d) Postoperative appearance showing the sector iridociclectomy and the pupilloplasty.

A 37-year-old woman presented herself at the Ophthalmological Department for a left eye pupil deformity. The slit lamp microscopy revealed an iris neoformation in the inferior temporal quadrant of the iris. This neoformation caused pupil dyscoria and corectopia and uveal ectropion. The color of the neoformation was light pink and it was richly vascularized (Figures 1(a) and 1(b)). The Ultrasound Biomicroscopy examination showed a neoformation localized in the iris stroma, undefined within the normal iris tissue, occupying the iris corneal angle, but not seeming to invade the ciliary body (Figure 1(c)).

Iris fluorescent angiography showed new formed vessels with abundant leakage in the neoformation.

After a complete workup, in the suspicion of an uveal amelanotic melanoma, surgical excision of the neoformation was programmed. Before surgery, a laser photocoagulation of the temporal inferior retinal quadrant was performed to prevent retinal detachment.

Under general anesthesia a sector iridociclectomy and a pupilloplasty were performed.

**Figure 2:**
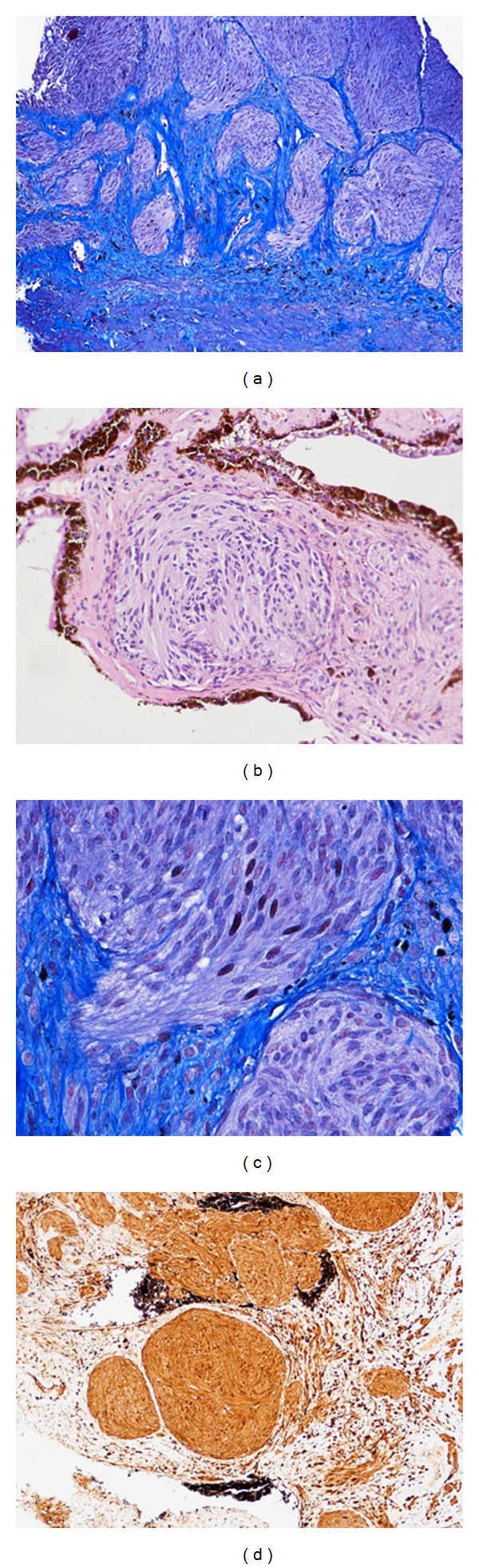
(a) Nests of unpigmented spindle cells in a fibrous stroma (Mallory stain, ×100). (b) Neoplastic cells infiltrate the iris stroma (H&E, ×200). (c) Uniform appearance of cellular morphology lacking in nuclear atypia and mitoses (Mallory stain, ×400). (d) Immunohistochemical staining with HMB45 confirms the melanocytic nature of the neoplastic cells (original magnification, ×100).

Histologically the lesion was composed of a dense and diffuse proliferation of amelanotic cells nested in a nevoid pattern and surrounded by a dense fibrous stroma (Figure 2(a)).

The cells varied in morphology from short spindle-shaped to dendritic one. The neoplastic proliferation involved the iris stroma and, focally, the contiguous ciliary body (Figure 2(b)). All cells showed uniform, oval, vesicular nuclei with finely dispersed chromatin and inconspicuous nucleoli. Neither mitotic figures, cellular atypia, or necrosis were present (Figure 2(c)). Immunohistochemical reactions were intensely positive for S-100 protein and HMB45 (Figure 2(d)), but negative for alfa smooth-muscle actin, desmin, and CD56. The MIB-1 labeling index was low (Ki67 less than 1%).

On the basis of morphologic and immunohistochemical features, the tumor was diagnosed as amelanotic cellular blue nevus.

## 3. Discussion

Blue nevus was first described by Tièche in 1906 [12]. Earlier authors [13, 14] described similar lesions as chromatophoroma and melanofibroma. The common blue nevus is a flat to slightly elevated, smooth surfaced macule, papule, or plaque that is gray-blue to bluish black in color. Lesions are usually solitary and found on the head and the neck, the sacral region, and the dorsal aspects of hands and feet. Two varieties of blue nevi are described: the common blue nevus and the cellular type [1]. In common blue nevus, a vaguely nodular collection of poorly melanized spindled melanocytes and deeply pigmented dendritic melanocytes within thickened collagen bundles is seen. Scattered melanophages are usually noted. No mitoses are present. In cellular blue nevus, a well-defined nodule formed by fascicles and nests tightly packed, moderately sized, spindle shaped, and melanocytes with scattered melanophages are visible. Blue nevi may be divided into the following types: epithelioid blue nevus (classic description is with the Carney complex [15], but also without this condition), atypical blue nevi, deep penetrating blue nevi, sclerosing blue nevi, and amelanotic blue nevi [16]. Amelanotic cellular blue nevi with spindle cells is an unusual variant of the common and cell-rich blue nevus with atypical clinical and pathologic appearance due to the lack of pigment [17].

Differential diagnosis included all nonmelanocytic tumors with spindle cells [18].

 In particular, amelanotic cellular blue nevus must be distinguished from malignant cellular blue nevus [19] and other variants of fusocellular melanomas, but also from mesectodermal leiomyoma [20], a rare benign tumor with double neurogenic and myogenic differentiation which takes origin from neural crests. The definitive diagnosis can be acquired with the aid of electron microscopy or with immunohistochemical study.

Our case is the first description of a blue amelanotic nevus involving the eye. Ophthalmologists must be aware of the possibility of this entity in the differential diagnosis of eye neoformations.
